# Exploring the Potential of *Ribes nigrum* L., *Aronia melanocarpa* (Michx.) Elliott, and *Sambucus nigra* L. Fruit Polyphenol-Rich Composition and Metformin Synergy in Type 2 Diabetes Management

**DOI:** 10.1155/2024/1092462

**Published:** 2024-06-10

**Authors:** Katarzyna Zima, Barbara Khaidakov, Laura Banaszkiewicz, Krzysztof Lemke, Paulina Karolina Kowalczyk

**Affiliations:** ^1^ Department of Physiology Medical University of Gdańsk Dębinki 1 80-211, Gdańsk, Poland; ^2^ R&D Department AronPharma Ltd. Trzy Lipy Street 3 80-172, Gdańsk, Poland

**Keywords:** diabetes, plant extracts, polyphenol

## Abstract

Type 2 diabetes, characterized by insulin resistance and impaired glucose homeostasis, is commonly managed through lifestyle interventions and medications such as metformin. Although metformin is generally well-tolerated, it may cause gastrointestinal adverse effects and, in rare cases, precipitate lactic acidosis, necessitating cautious use in individuals with renal dysfunction. Additionally, concerns regarding its impact on hepatic function have led to its discontinuation in cirrhotic patients. This study explores the potential synergistic benefits of a polyphenol-rich blend containing black currant, chokeberry, and black elderberry extracts alongside metformin in managing type 2 diabetes. In vitro results highlighted distinct effects of AMPK pathway modulation, showcasing reductions in cholesterol and triglyceride levels alongside a notable enhancement in glucose uptake. The blend, when combined with metformin, significantly reduced insulin levels and fasting glucose concentrations in an in vivo model. Furthermore, hepatic analyses unveiled a modulation in cellular pathways, suggesting a potential influence on lipid metabolism, attenuation of inflammatory pathways, a decrease in cellular stress response, and antioxidant defense mechanisms, collectively implying a potential reduction in liver fat accumulation. The findings suggest a potential complementary role of polyphenols in enhancing the efficacy of metformin, possibly allowing for reduced metformin dosage and mitigating its side effects. Further clinical studies are warranted to validate these findings and establish the safety and efficacy of this nutraceutical approach in managing type 2 diabetes.

## 1. Introduction

Type 2 diabetes (T2D) is a prevalent chronic metabolic disorder characterized by insulin resistance and impaired glucose homeostasis [1]. Based on the 2019 report from the International Diabetes Federation Diabetes Atlas, an estimated 463 million adults were living with diabetes globally, with projections suggesting this number will rise to 578 million by 2030 and 700 million by 2045, highlighting the escalating global prevalence of T2D [2]. Current treatment strategies for T2D include lifestyle interventions and medication, such as metformin, to control blood sugar levels. Metformin is a widely used medication for the treatment of T2D [3]. Metformin's mechanism of action in T2D patients is essentially based on the reduction of hepatic glucose synthesis and the improvement of insulin sensitivity. It activates AMP-activated protein kinase (AMPK), an important regulator of cellular energy homeostasis, resulting in decreased gluconeogenesis in the liver [4]. Furthermore, metformin enhances glucose absorption and use by peripheral tissues, notably muscle cells, decreasing blood glucose levels without increasing insulin secretion. Metformin is an excellent first-line treatment for T2D since it lowers blood glucose levels, promotes weight loss, and has a minimal risk of inducing hypoglycemia [5]. It is generally associated with favorable tolerability; however, similar to any pharmacological intervention, it is not devoid of potential adverse effects. The most common adverse effects of metformin are gastrointestinal, including diarrhea, nausea, and vomiting. Up to 30% of people using metformin experience these symptoms [6]. Nevertheless, these symptoms commonly exhibit resolution with the passage of time and can be effectively mitigated through dosage modifications or adjunctive pharmacotherapy. In rare instances, metformin administration may precipitate lactic acidosis, a grave metabolic derangement with potential life-threatening consequences if not promptly addressed [7]. This phenomenon is more likely to manifest in patients exhibiting renal dysfunction or comorbidities predisposing them to lactic acidosis, thereby necessitating the cautious employment of metformin in such individuals [8]. Additionally, metformin has elicited apprehension regarding its impact on hepatic function. While studies have suggested a potential protective effect of metformin against the development of liver cancer, its administration commonly ceased following the diagnosis of cirrhosis due to concerns over heightened susceptibility to metformin-associated adverse effects in patients with compromised hepatic function [9]. Therefore, alternative or adjunct therapies are being explored to enhance the effectiveness of existing treatments. In this regard, polyphenol-rich fruits have emerged as promising candidates due to their reported health benefits, including potential antidiabetic effects [10].

Polyphenols are bioactive compounds found in plants, known for their antioxidant and anti-inflammatory properties. Evidence from numerous studies suggests that polyphenol-rich fruits may support the metformin treatment for T2D [11–13]. Among the various types of them, black currant (*Ribes nigrum* L., RN), chokeberry (*Aronia melanocarpa* (Michx.) Elliott; AM), and black elderberry (*Sambucus nigra* L. SN) fruits have gained attention as potential adjuncts to conventional treatments, such as metformin, in the management of T2D [14–16]. These fruits exhibit diverse bioactive profiles, including high concentrations of anthocyanins [17, 18]. Additionally, their total phenolic content, antioxidant activity, and functionality contribute to their potential therapeutic benefits [19, 20].

Polyphenols have been shown to modulate carbohydrate metabolism by restoring beta cell integrity and physiology, enhancing insulin release, and improving glucose utilization [21]. RN anthocyanins, in particular, have been found to activate endothelial nitric oxide synthase and induce vasodilatory function, potentially improving cardiovascular health in individuals with diabetes [22]. Furthermore, polyphenol-rich sources have been associated with a decreased risk of T2D [23]. AM, specifically, has been demonstrated to possess a range of desirable properties, including antioxidant, anti-inflammatory, antiproliferative, and antimicrobial properties [24]. These attributes make it a promising candidate for managing the oxidative stress and chronic inflammation associated with T2D. In vitro studies have highlighted the antioxidant activity of extracts from polyphenol-rich fruits, as well as their ability to inhibit key enzymes involved in hyperglycemia and hypertension [25]. It was previously shown that supplementation with SN extract enhanced diabetes management in rodents. Insulin resistance was reduced by both polar and lipophilic extracts in diabetic rats fed a high-fat diet [26]. SN extract was also found to reduce liver weight, triglyceride concentration, insulin resistance, and fatty acid synthase mRNA in the liver [27]. Anthocyanin-rich extracts derived from AM have demonstrated cardioprotective effects, primarily due to their potent antioxidant properties [28]. These findings suggest that incorporating these fruits or their extracts into the diet may help regulate glucose levels and reduce the risk of complications, for example, cardiovascular. This study is aimed at determining the effect of an AP029 blend containing RN, AM, and SN on the cellular mechanisms involved in diabetes.

## 2. Materials and Methods

### 2.1. Blend Preparation

The RN, AM, and SN extracts were provided by Greenvit Ltd. Pure extracts were produced by water/ethanol or water extraction in mild conditions in order to preserve thermolabile compounds. The liquid extracts were concentrated in a vacuum and spray-dried. Pure extracts were standardized.

For total phenolic concentration (TPC) determination, two extract samples were initially prepared by diluting them in a methanol–water solution (4:6 *v*/*v*) until a concentration of 1 mg/mL was reached. Subsequently, 1 mL of each diluted sample was combined with 4 mL of water and 0.5 mL of Folin–Ciocalteu reagent (Sigma-Aldrich, Saint Louis, MO, USA). Following a 30-min incubation period at room temperature (RT) in the dark, the absorbance of the samples at 760 nm was measured using a NanoPhotometer® NP80 spectrophotometer (Implen GmbH, München, Germany). To establish TPC, a 6-point calibration curve with known concentrations of caffeic acid (Sigma-Aldrich, Saint Louis, MO, USA) as the standard was created. The concentration of polyphenols in the samples was determined by comparing their absorbance to this calibration curve, typically expressed in terms of caffeic acid equivalents (CAEs) per unit volume (e.g., mg CAE/mL).

For anthocyanin concentration determination, two extract samples were initially diluted in a methanol–water mixture (4:6 *v*/*v*) to achieve a concentration of 0.1 mg/mL. These diluted samples were then further diluted with pH 1.0 and pH 4.5 buffers in 10-mL volumetric flasks. After being allowed to sit at RT in the dark for 5 min, their absorbance at specific wavelengths, 520 nm and 700 nm, was measured using the NanoPhotometer® NP80 spectrophotometer. Anthocyanin concentration was calculated using the molecular weight of cyanidin-3-glucoside (C-3-Glu) and the molar absorbance coefficient at these wavelengths, following the principles of the Beer–Lambert law.

### 2.2. Chromatographic Analysis

After optimization of the chromatographic conditions, the subsequent working conditions were used: samples of the blend were diluted in a methanol–water mixture (2:8 *v*/*v*) with an addition of 2% formic acid to achieve a concentration of 1 mg/mL. Before the UPLC analysis, these diluted samples were injected through a syringe filter. The concentration of the standard solution was 1 mg/mL as well. Next, 1 *μ*L of the sample was injected into the UPLC–PDA system.

All analyses were performed on an ACQUITY UPLC I-Class PLUS System with a PDA detector (Waters, Milford, MA, USA). Chromatographic separation was achieved on a Waters ACQUITY UPLC® BEH C18 column (1.7 *μ*M 130 Å, 100 mm × 2.1 mm) with an ACQUITY UPLC BEH C18 VanGuard Pre-column (1.7 *μ*M 130 Å, 5 mm × 2.1 mm). The column was maintained at 40°C and the autosampler at 5°C. The mobile phase consisted of 2% formic acid in water (*v*/*v*, component A) and ACN (component B). The purities for all of the compounds and solvents were all above 98% by HPLC analysis.

The mobile phase was eluted under the following gradient conditions in relation to component A: initial, 96.0%; 3.00 min, 96.0%; 6.00 min, 85.0%; 7.00 min, 83.0%; 10.00 min, 20.0%; 10.10 min, 96.0%; 13.00 min, 96.0%. The flow rate was set at 0.25 mL/min. The total analysis run time was 15 min.

PDA acquisitions were carried out within the range of 200–700 nm, specifically at wavelengths of 520 nm and 535 nm with a resolution of 4.8 nm. The peak purity of the analytes was verified by comparing the retention time and UV spectrum to those of the pure standards.

The method was validated before use according to the guidelines of the International Council for Harmonization (ICH) [29] and the Food and Drug Administration (FDA) [30]. All validation parameters met the requirement criteria. Data acquisition, result analysis, and processing were performed using Empower Software Solutions by Waters.

### 2.3. Antioxidant Capacity of AP029 Blend

#### 2.3.1. Oxygen Radical Antioxidant Capacity (ORAC) Assay

The assessment of ORAC for the AP029 blend was conducted in accordance with the manufacturer protocol (Abcam, Cambridge, UK). The trolox (Sigma-Aldrich, Saint Louis, MO, USA) standard curve (ranging from 0 to 50 *μ*M) was established. Twenty-five microliters of the trolox standard or samples was added to a 96-well plate, followed by the addition of 150 *μ*L of the fluorescein solution to each well. After thorough mixing, the plate was incubated for 30 min at 37°C. Subsequently, 25 *μ*L of the Free Radical Initiator Solution was added to each well. Fluorescence readings were recorded by the Perkin Elmer EnVison 2103 Multilabel Reader (Perkin Elmer, Waltham, MA, USA), with an excitation wavelength of 485 nm and emission at 530 nm, with readings taken in increments of 5 min for a total of 60 min. The oxygen radical absorbance capacity (ORAC) value for the AP029 was expressed in millimoles of trolox per gram.

#### 2.3.2. DPPH Assay

A solution with a concentration of 1 mg/mL of the trolox standard (Sigma-Aldrich, Saint Louis, MO, USA) was prepared in methanol. Serial dilutions of the standard were prepared at concentrations ranging from 3.125 to 500 *μ*g/mL. A solution containing 1 mg/mL of AP029 in methanol:H_2_O (in a volumetric ratio of 1:1) was made. Serial dilutions of AP029 were prepared at concentrations ranging from 5 to 500 *μ*g/mL. In a 96-well plate, 100 *μ*L of a 0.25 mM 2,2-diphenyl-1-picrylhydrazyl (DPPH) solution (Sigma-Aldrich, Saint Louis, MO, USA) in methanol was added to each well. Subsequently, 100 *μ*L of the trolox and AP029 solutions were added to each DPPH-filled well. The plate was then incubated for 30 min at 25°C and 400 rpm. Absorbance was measured at 520 nm using a Perkin Elmer EnVison 2103 Multilabel Reader (Perkin Elmer, Waltham, MA, USA). Results were expressed as % DPPH inhibition, calculated as %inhibition = 1 − ((average absorbance of the test substance)/(average absorbance of DPPH) × 100%). Additionally, a standard curve for trolox was used to determine the concentration of the test substance inhibiting DPPH by 50%.

#### 2.3.3. Ferric Reducing Antioxidant Power (FRAP) Assay

The assessment of FRAP for the AP029 blend was conducted in accordance with the manufacturer protocol (Abcam, Cambridge, UK). Briefly, serial dilutions of the ferrous standard were prepared at concentrations ranging from 0 to 20 nmol/well. Serial dilutions of AP029 were prepared at concentrations ranging from 5 to 500 *μ*g/mL. One hundred ninety microliters of reaction mix (containing assay buffer, probe, and FeCl_3_ solution) was added to a 96-well plate together with 10 *μ*L of standard or sample per well. Absorbance was measured at 594 nm using a Perkin Elmer EnVison 2103 Multilabel Reader (Perkin Elmer, Waltham, MA, USA). The following calculations were used to determine the mM Ferrous Equivalents of the samples: Sample FRAP = ferrous ammonium sulfate amount from standard curve (nmol) × (sample volume added in the sample wells (*μ*L)/sample volume added in the sample wells (*μ*L)).

### 2.4. In Vitro Cell Cultures

#### 2.4.1. 3T3-L1 Cell Culture and Differentiated Adipocyte Model

Fibroblasts derived from Swiss albino mouse embryo, 3T3-L1, were obtained from the American Type Culture Collection (ATCC; Manassas, VA, USA) and cultured in Dulbecco's Modified Eagle Medium (DMEM; Biowest, Nuaillé, France) supplemented with 10% bovine calf serum (BCS; Sigma-Aldrich, Saint Louis, MO, USA) and 1% penicillin–streptomycin (Pen–Strep; Sigma-Aldrich, Saint Louis, MO, USA) and allowed to reach 70% confluency in a humidified atmosphere of 5% CO_2_ at 37°C. Next, cells were induced to differentiate into adipocytes using a standard protocol, which involved treating the cells with a differentiation cocktail containing DMEM supplemented with 10% fetal bovine serum (FBS; Biowest, Nuaillé, France), 1 *μ*M dexamethasone (Sigma-Aldrich, Saint Louis, MO, USA), 0.5 mM 3-isobutyl-1-methylxanthine (IBMX; Sigma-Aldrich, Saint Louis, MO, USA), and 1 *μ*g/mL insulin (Sigma-Aldrich, Saint Louis, MO, USA) for 48 h. After this time, the medium was changed into an adipocyte maintenance cocktail, including DMEM supplemented with 10% FBS and 1 *μ*g/mL insulin. Fourteen days after differentiation induction, cells were fixed in 4% paraformaldehyde (PFA; Sigma-Aldrich, Saint Louis, MO, USA) for 15 min at RT, stained with 0.2% Oil Red O (Sigma-Aldrich, Saint Louis, MO, USA) for 30 min at RT, and washed 5× with distilled water (dH2O). To observe lipid accumulation, a sign of adipocyte development, light microscopy was performed (IB-100; Delta Optical, Gdansk, Poland) (Figure S1).

#### 2.4.2. HepG2 Cell Culture and Insulin Resistance Model

Human hepatoma HepG2 cells were obtained from the ATCC and cultured in glucose-free Minimum Essential Medium (MEM; Biowest, Nuaillé, France) supplemented with 10% FBS and 1% Pen–Strep, containing 5.5 mM D-glucose in a humidified atmosphere of 5% CO_2_ at 37°C. HepG2 cells were grown to 70% confluence and then kept in serum-free MEM overnight before being used in the experiments. Cells were then cultured for a further 24 h in serum-free MEM containing either normal (5.5 mM) or high (30 mM) concentrations of D-glucose (Sigma-Aldrich, Saint Louis, MO, USA) and stimulated with 100 nM insulin for 10 min before collection. The in vitro model's accuracy was determined by western blot (Figure S2 A).

#### 2.4.3. Thle-2 Cell Culture and Insulin Resistance Model

Thle-2 were derived from primary normal liver cells (by infection with SV40 large T antigen cells), obtained from the ATCC, and cultured on plates coated with type I collagen (Advanced BioMatrix, Carlsbad, CA, USA) in Bronchial Epithelial Cell Growth Basal Medium (BEBM; Lonza, Basel, Switzerland) supplemented with 0.4% BPE, 0.1% hydrocortisone, 0.1% retinoic acid, 0.1% transferrin, 0.1% triiodothyronine, 0.1% hEGF (BEGM; Lonza, Basel, Switzerland), and 1% Pen–Strep, containing 6 mM D-glucose, in a humidified atmosphere of 5% CO_2_ at 37°C. Cells were grown to 70% confluence before being used in the experiments. Cells were then cultured for a further 24 h in BEGM supplemented with 2% fatty acid-free BSA (Sigma-Aldrich, Saint Louis, MO, USA), containing 1 mM of oleic acid (Sigma-Aldrich, Saint Louis, MO, USA) and 1 mM of palmitic acid (Sigma-Aldrich, Saint Louis, MO, USA), mixed in a 2:1 ratio. Subsequently, the cells were incubated in the presence of either normal (5.5 mM) or high (30 mM) concentrations of D-glucose for the next 24 h and stimulated with 100 nM insulin for 10 min before collection. The in vitro model's accuracy was determined by western blot (Figure S2 B).

### 2.5. Glucose Uptake Study

Differentiated 3T3-L1 adipocytes were cultured in 96-well plates and subjected to an overnight starvation period without FBS. Following this, the cells were exposed to glucose-free DMEM supplemented with L-glutamine (Biowest, Nuaillé, France) +2% bovine serum albumin (BSA; BioShop Canada Inc., Burlington, Canada) for 1 h of 5% CO_2_ at 37°C. The cells were then stimulated with 100 nM insulin, 1 mM metformin (ChemScene, NJ, USA), 100 *μ*g/mL AP029, and a combination of 100 *μ*g/mL AP029 with 1 mM metformin for 30 min of 5% CO_2_ at 37°C. To assess glucose uptake, a fluorescently labeled deoxyglucose analog (2-NBDG; Abcam, Cambridge, UK) was introduced at a final concentration of 10 *μ*M/well in glucose-free DMEM supplemented with L-glutamine for a period of 1 h of 5% CO_2_ at 37°C. Next, the cells were then washed 3× with PBS and suspended in 200 *μ*L of PBS/well. The spectrophotometric reading was taken at excitation/emission = 485/535 nm on the Perkin Elmer EnVison 2103 Multilabel Reader (Perkin Elmer, Waltham, MA, USA).

### 2.6. Determination of Triglycerides and Cholesterol Contents

The Cholesterol/Cholesterol Ester-Glo Assay kit (Promega, Madison, WI, USA) was used to determine cholesterol levels in the HepG2 and Thle-2 cells, activated as described in points 3.2 and 3.3, respectively, and cultured for the next 24 h with simultaneous treatment with 2 mM metformin, 100 *μ*g/mL AP029, and a combination of 100 *μ*g/mL AP029 with 2 mM metformin. The assay was performed following the manufacturer's instructions. Analogous measurements of triglyceride levels were made in HepG2 and Thle-2 cells using the Triglyceride-Glo™ Assay kit (Promega, Madison, WI, USA), following the manufacturer's instructions. The luminescence signal was measured utilizing a Perkin Elmer EnVison 2103 Multilabel Reader (Perkin Elmer, Waltham, MA, USA).

### 2.7. Animal Study

A total of 40 male diabetic BKS(D)-Lepr^db^/JOrlRj (db/db) mice (7 weeks old) were purchased from Janvier Labs, France. The animals were quarantined for 6 days after shipping, and then the mice were left to acclimatize for 7 days before the start of the experiment. Mice were housed in two mice per cage in a temperature-controlled (21 ± 2°C) room on a 12:12 light/dark cycle. Mice were randomly divided into four groups, including the control group (*n* = 10), the metformin group (*n* = 10), the AP029 group (*n* = 9, one mouse was excluded during the study because of an abscess), and the AP029 + metformin group (*n* = 10). Mice were fed a standardized mouse diet and given sterilized drinking water ad libitum.

Animal experiments were conducted in the Centre of Experimental Medicine, Medical University of Bialystok, and were approved by the Local Ethical Committee for Animal Experiments in Olsztyn (no. 24/2022). The control group received a solution of 0.9% NaCl, the metformin group received metformin 10 mg/mL in 0.9% NaCl, the AP029 group received polyphenolic composition 5 mg/mL in 0.9% NaCl, and the AP029 + metformin group received composition 5 mg/mL together with metformin 10 mg/mL in 0.9% NaCl. Each group of mice received an intragastric solution of 1 mL/100 g body weight/day for 6 weeks.

### 2.8. Protein Level Analysis

#### 2.8.1. Lysate Preparation From Cell Culture

HepG2 cells were washed twice with cold phosphate-buffered saline (PBS; Biowest, Nuaillé, France) and scraped off the culture dish before centrifugation at 12,000 g for 15 min at 4°C to obtain a cell pellet. Then, the cell pellet was lysed using RIPA buffer (50 mM Tris HCl, pH 8.0; 5 mM EDTA, pH 8.0; 1% NP-40; 150 mM NaCl; 0,5% sodium deoxycholate; 0.1% SDS) with protease and phosphatase inhibitors (Roche, Basel, Switzerland) and incubated on ice for 30 min (vortexing every 5 min), and the cell debris was removed by centrifugation at 12,000 g for 15 min at 4°C. The supernatant was then transferred to a fresh tube and stored at −80°C until further use.

#### 2.8.2. Lysate Preparation From Mouse Tissues

The 50 mg piece of animal tissue was excised on dry ice, followed by precise weighing of the samples to ensure accuracy, subsequent mincing of the tissue into small pieces using a scalpel, and transfer to a tube. Six hundred microliters of RIPA lysis buffer with protease and phosphatase inhibitors was added to each tube to initiate tissue lysis. The tubes were subsequently incubated on ice for 2 h with vortexing every 15 min to promote adequate lysis. After incubation, the tubes were subjected to centrifugation at 16,000 g for 20 min at 4°C. The supernatant was collected, transferred to a fresh tube, and stored at −80°C until further use.

#### 2.8.3. Protein Quantification

Protein concentrations in the lysates were determined using the Bradford reagent (Bio-Rad, Hercules, CA, USA) according to the manufacturer's instructions. Briefly, 10 *μ*L of each lysate was mixed with 200 *μ*L of Bradford reagent, and the absorbance was measured at 595 nm using a microplate reader (Perkin Elmer, Waltham, MA, USA). BSA in the range of 0.0–1.0 mg/mL was used as a standard. The lysates were equalized in final concentration to 3 *μ*g/mL.

#### 2.8.4. Western Blot

Equal amounts of protein lysates were separated by SDS-PAGE using 10% polyacrylamide gels and transferred onto polyvinylidene difluoride membranes (PVDF; Sigma-Aldrich, Saint Louis, MO, USA). The membranes were blocked with 5% BSA in TBST buffer (20 mM Tris-HCl, pH 7.5, 150 mM NaCl, and 0.1% Tween-20) for 2 h at RT. The membranes were then incubated with primary antibodies against the following proteins: acetyl-CoA carboxylase (ACC; #3662, Cell Signaling, Danvers, MA, USA), phospho-ACC (pACC; #11818, Cell Signaling, Danvers, MA, USA), protein kinase B (Akt; #9272S, Cell Signaling, Danvers, MA, USA), phospho-Akt (pAkt; #sc-514032, Santa Cruz Biotechnology, Dallas, TX, USA), AMPK (#2532, Cell Signaling, Danvers, MA, USA), phospho-AMPK (pAMPK; #2535, Cell Signaling, Danvers, MA, USA), glycogen synthase kinase 3 (GSK-3; #ab227208, Abcam, Cambridge, UK), phospho-GSK-3 (pGSK-3; #9331S, Cell Signaling, Danvers, MA, USA), 3-hydroxy-3-methylglutaryl-CoA reductase (HMGCR; #FNab03929, FineTest, Wuhan, Hubei, China), phospho-HMGCR (pHMGCR; #bs-4063R, BiossAntibodies, Woburn, MA, USA), sirtuin 1 (SIRT1; #9475, Cell Signaling, Danvers, MA, USA), sterol regulatory element-binding protein 1 (SREBP1; #sc-365513, Santa Cruz Biotechnology, Dallas, TX, USA), superoxide dismutase 2 (SOD2; #sc-133134, Santa Cruz Biotechnology, Dallas, TX, USA), transcription factor of the nuclear factor *κ*B (NF-*κ*B/p65; #8242, Cell Signaling, Danvers, MA, USA), and phospho-NF-*κ*B/pp65 (pNF-*κ*B/pp65; #3033, Cell Signaling, Danvers, MA, USA) overnight at 4°C. After washing with TBST buffer, the membranes were incubated with secondary antibodies conjugated with horseradish peroxidase (HRP): anti-rabbit IgG (#7074S, Cell Signaling, Danvers, MA, USA) and anti-mouse IgG (#ab6728, Abcam, Cambridge, UK) for 2 h at RT. The protein bands were visualized using Pierce ECL Western Blotting Substrate (Thermo Fisher Scientific, Waltham, MA, USA) and imaged using the Azure 280 Imaging System (Azure Biosystems, Dublin, CA, USA). Densitometry was performed using Quantity One 1-D Analysis Software (Bio-Rad, Hercules, CA, USA).

### 2.9. Statistical Analysis

GraphPad Prism 9.5.0 software was used to analyze the data. The experimental results were expressed as mean ± standard deviation. The normality of the data was assessed using the Shapiro–Wilk test. Subsequently, statistical analyses were conducted based on the distribution of the data. Parametric analyses for normally distributed data comprise the utilization of Student's *t*-test or one-way analysis of variance (ANOVA), followed by post hoc testing to discern statistical differences. In cases where normality assumptions were violated, nonparametric tests were invoked (Mann–Whitney *U* or Kruskal–Wallis test). *p* < 0.05 was considered significant.

## 3. Results

### 3.1. Blend Composition

**Figure 1:**
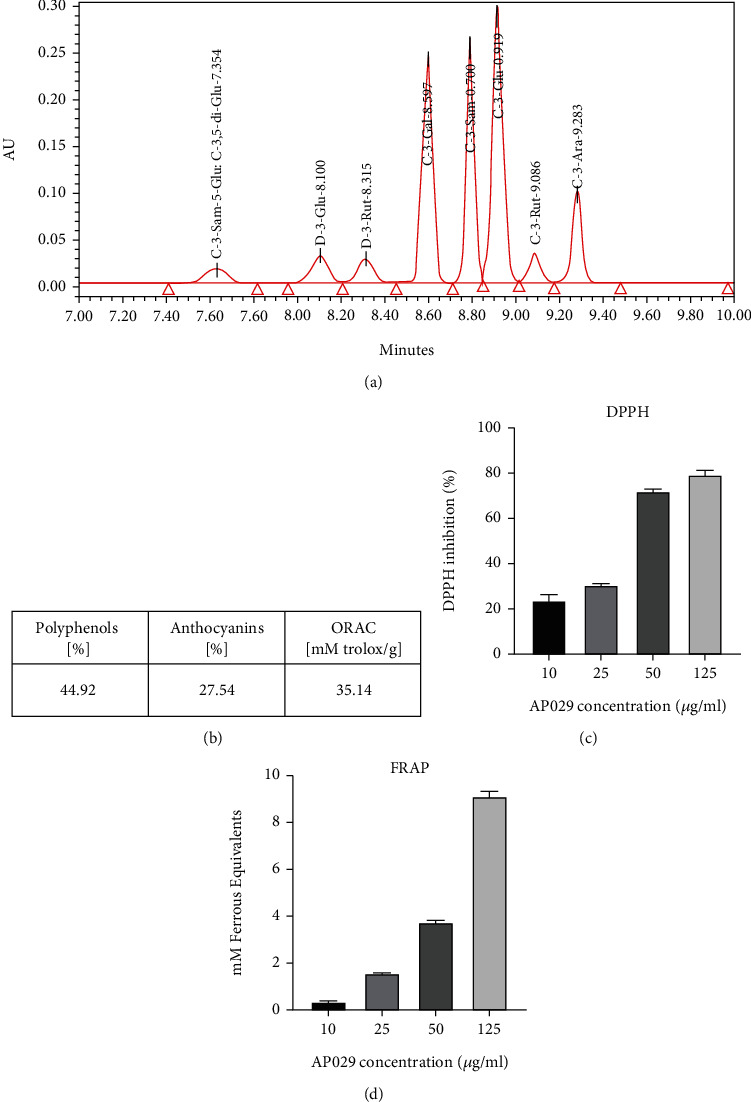
(a) The chromatogram of the AP029 with identified anthocyanin compounds. (b) The total phenolic content was 44.92% (m/m) quantified based on the Folin-Ciocalteu assay with caffeic acid as a calibrating curve. The anthocyanin content in the blend was determined to be 27.54% (m/m) using UV-Vis spectroscopy. Oxygen radical absorbance capacity (ORAC) value for the AP029 was expressed in millimoles of trolox per gram. Concentration-dependent antioxidant activity was measured by (c) the 2,2-diphenyl-1-picrylhydrazyl (DPPH) assay and (d) the ferric reducing antioxidant power (FRAP) assay.

The chromatographic analysis for the blend showed the presence of compounds such as cyanidin-3-O-sambubioside-5-O-glucoside (C-3-Sam-5-Glu), cyanidin-3,5-O-diglucoside (C-3,5-di-Glu), delphinidin 3-O-glucoside (D-3-Glu), delphinidin 3-O-rutinoside (D-3-Rut), cyanidin 3-O-galactoside (C-3-Gal), cyanidin 3-O-sambubioside (C-3-Sam), cyanidin 3-O-glucoside (C-3-Glu), cyanidin 3-rutoside (C-3-Rut), cyanidin 3-O-arabinoside (C-3-Ara), and cyanidin 3-xyloside (C-3-Xyl) (Figure 1(a)). The total phenolic content was quantified based on the Folin–Ciocalteu assay with caffeic acid as a calibrating curve. The phenolic content was 58.1% (m/m) (Figure 1(b)). Utilizing UV-Vis spectroscopy, the anthocyanin content within the blend was quantified to be 27.54% (m/m) (Figure 1(b)). ORAC measurements were established as part of a complete investigation of the antioxidant properties of the AP029 blend. The levels, measured in millimoles of trolox per gram, were found to be significantly high, at 35.14 mM trolox/g (Figure 1(b)). The AP029 blend's concentration-dependent antioxidant activity was determined using the DPPH assay. The AP029 mix inhibited DPPH at different concentrations, with 79.4% at 125 *μ*g/mL, 72.0% at 50 *μ*g/mL, 30.6% at 25 *μ*g/mL, and 23.7% at 10 *μ*g/mL (Figure 1(c)). The FRAP assay was used to determine the antioxidant activity of the AP029 blend. The Ferrous Equivalents recorded a concentration-dependent response, with values of 0.36, 1.56, 3.75, and 9.13 mM at concentrations of 10, 25, 50, and 125 *μ*g/mL of the AP029 blend, respectively (Figure 1(d)).

### 3.2. Synergistic Effect of AP029 and Metformin on Glucose Uptake in 3T3-L1 Adipocytes

**Figure 2:**
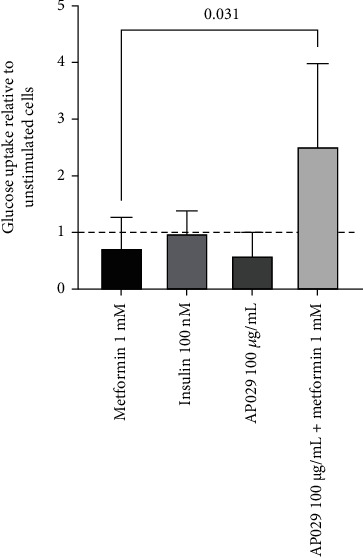
Effect of glucose uptake on differentiated adipocytes of the 3T3-L1 cell line stimulated with insulin (100 nM), metformin (1 mM), AP029 (100 *μ*g/mL), and a combination of AP029 (100 *μ*g/mL) with metformin (1 mM). The graph shows mean relative values ± SD with respect to unstimulated cells. Comparisons with *p* < 0.05 were displayed.

Glucose uptake analysis was performed on differentiated 3T3-L1 adipocytes. The confirmation of successful differentiation was verified using Oil Red O staining, as shown in Figure S1. The effect of AP029 on glucose uptake in 3T3-L1 adipocytes was investigated in the presence and absence of metformin and insulin. According to the findings, the uptake of glucose by 3T3-L1 adipocytes was not impacted by insulin, metformin, or AP029 when used alone. The combination of AP029 and metformin resulted in a synergistic effect, with glucose uptake increasing by 3.44-fold compared to the metformin control (2.53 (SD 1.44); *p* = 0.0307) (Figure 2).

### 3.3. AP029 Mode of Action: Modifying the AMPK Pathway in the In Vitro Insulin Resistance Model of HepG2 Hepatocytes

**Figure 3:**
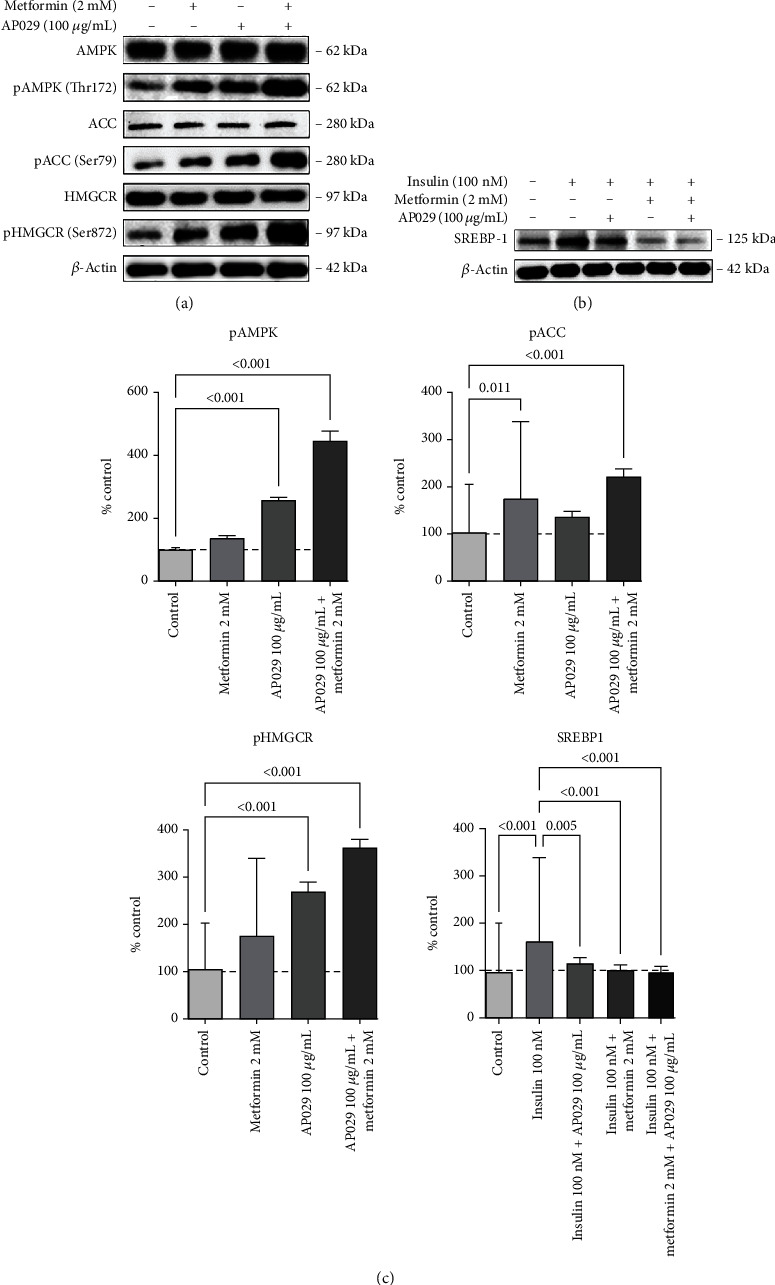
Representative western blot images showing the level of phosphorylation of (a) AMPK, ACC, and HMGCR proteins and (b) SREBP1 protein level in HepG2 cells cultured in the presence of metformin (2 mM), AP029 (100 *μ*g/mL), and AP029 (100 *μ*g/mL) with metformin (2 mM). (c) Densitometric analyses were normalized to *β*-actin and presented as a percentage of the 30 mM glucose control. Data represent mean ± SD. Comparisons with *p* < 0.05 were displayed.

The results showed an increase in AMPK phosphorylation in the culture treated with metformin (134.2% (SD 7.9%); ns), AP029 (257.1% (SD 8.1%); *p* = 0.0006), and AP029 and metformin (447.3% (SD 43.1%); *p* < 0.0001) compared to the control culture without metformin, which served as the reference with a baseline phosphorylation level set at 100% (SD 4.6%) (Figures 3(a) and 3(c)). Additionally, the phosphorylation of ACC exhibited a 60.7% (160.7% (SD 15.8%); *p* = 0.0004) increase in the presence of metformin, a 36.2% (136.2% (SD 2.9%); *p* = 0.0144) increase in the presence of the AP029, and a 138.9% (238.9% (SD 17.0%); *p* < 0.0001) increase when cultured with the AP029 and metformin, relative to the control at 100% (SD 4.6%) (Figures 3(a) and 3(c)). The phosphorylation of HMGCR, which reduces cholesterol production, was found to increase by 64.3% (164.3% (SD 11.2%); ns) with metformin, a 157.7% (257.7% (SD 39.7%); *p* = 0.0003) increase in the presence of the AP029, and by 258.3% (358.3% (SD 21.6%); *p* < 0.0001) with the AP029 and metformin, compared to the control at 100% (SD 4.6%) (Figures 3(a) and 3(c)). The SREBP1 protein, known for its role in regulating de novo lipid production in the liver, exhibited changes in response to insulin and metformin. High insulin concentrations (100 nM) led to an increase of 66.5% (166.5% (SD 9.1%); *p* < 0.0001) in SREBP1 levels compared to 100% control (SD 3.4%), while metformin with insulin resulted in a decrease by 59.6% (106.9% (SD 5.8%); *p* < 0.0001) compared to insulin only. The addition of the AP029 with metformin in the in vitro culture showed a marginal decrease in SREBP1 protein levels, returning them to the level of the negative control of the metformin composition (100.8% (SD 3.7%); *p* < 0.0001) (Figures 3(b) and 3(c)).

### 3.4. Evaluation of the Effect of the AP029 on the Level of Cholesterol and Triglycerides in the In Vitro Insulin Resistance Model of HepG2 and Thle-2 Hepatocytes

**Figure 4:**
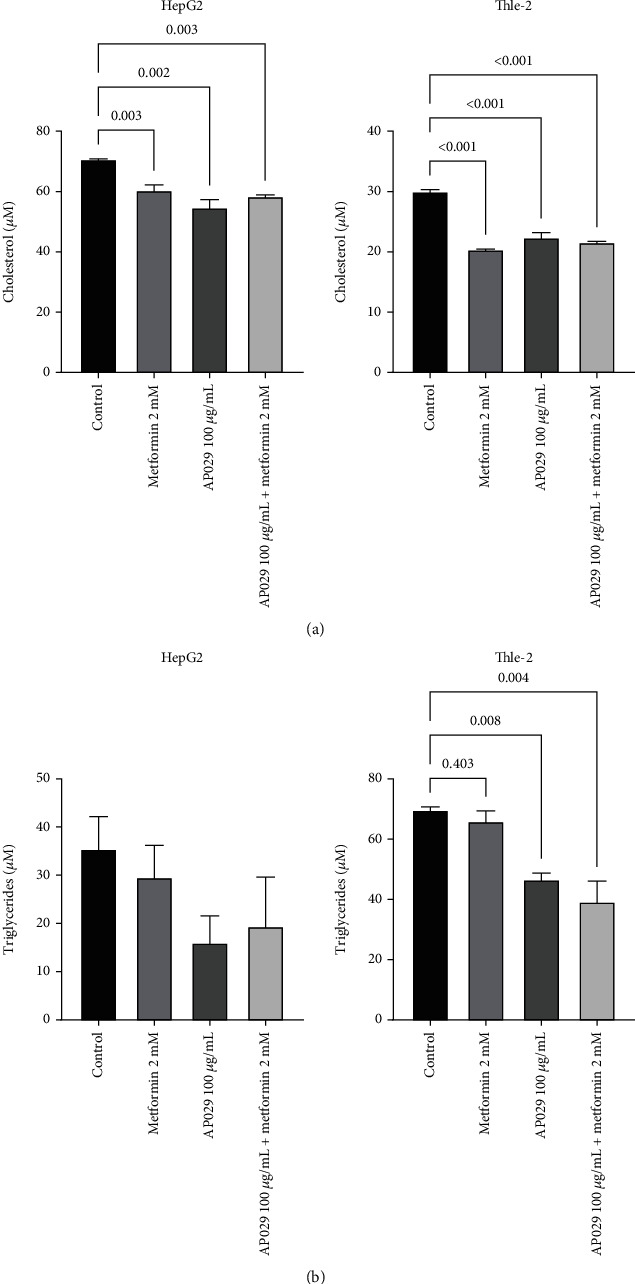
Effect of metformin (2 mM), AP029 (100 *μ*g/mL), and AP029 (100 *μ*g/mL) with metformin (2 mM) and control (30 mM glucose) on (a) cholesterol levels in the HepG2 and Thle-2 and (b) triglycerides in the HepG2 line and Thle-2 cell lines. Data represent mean ± SD. Comparisons with *p* < 0.05 were displayed.

The effects of metformin, the composition, and the composition with metformin are comparable in the HepG2 and Thle-2 lines. From a baseline level in the control 70.82 *μ*M (SD 0.1 *μ*M), the cholesterol level in the HepG2 line decreases to 60.49 *μ*M (SD 1.73 *μ*M; *p* = 0.0094), 54.8 *μ*M (SD 2.57 *μ*M; *p* = 0.0018), and 58.47 *μ*M (SD 0.44 *μ*M; *p* = 0.0049), in cultures with metformin, AP029, and AP029 with metformin, respectively (Figure 4(a)). In addition, in cultures conducted with metformin, AP029, and AP029 plus metformin, the cholesterol level in the Thle-2 line was 30.04 *μ*M (SD 0.3 *μ*M) for control and 20.42 *μ*M (SD 0.08 *μ*M; *p* < 0.0001), 22.43 *μ*M (SD 0.74 *μ*M; *p* = 0.0002), and 21.65 *μ*M (SD 0.1 *μ*M; *p* = 0.0001) for cultures with metformin, AP029, and AP029 with metformin, respectively (Figure 4(a)). Metformin treatment lowers the level of triglycerides in HepG2 line cells to 29.67 *μ*M (SD 6.53 *μ*M; ns) and in combination with AP029 to 19.46 *μ*M (SD 10.18 *μ*M; ns) compared to control 35.47 *μ*M (SD 6.7 *μ*M). The triglyceride level was decreased to 16.04 *μ*M (5.56 *μ*M; ns) by using the composition alone (Figure 4(b)). The Thle-2 line's triglyceride levels lowered substantially after administering metformin (66.05 *μ*M, SD 3.3 *μ*M; ns). The AP029 composition exhibits a noticeable effect, reducing triglyceride levels to 46.65 *μ*M (SD 2.12 *μ*M; *p* = 0.0142) and 39.31 *μ*M (SD 6.7 *μ*M; *p* = 0.0052) when combined with metformin (Figure 4(b)).

### 3.5. Evaluation of the Effect of AP029 Composition on Serum Glucose and Insulin Levels in the db/db Mouse Model

**Figure 5:**
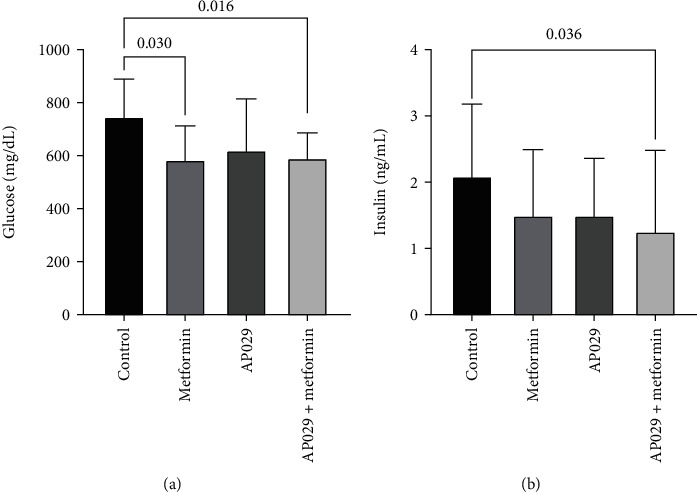
Effect of metformin (100 mg/kg b.w./day), AP029 (50 mg/kg b.w./day), and AP029 (50 mg/kg b.w./day) with metformin (100 mg/kg b.w./day) on (a) glucose and (b) insulin levels in serum of db/db mice. Data represent mean ± SD. Comparisons with *p* < 0.05 were displayed.

Serum insulin and glucose levels were measured at the end of the study. The results showed that the AP029 composition with metformin significantly reduced serum glucose levels (592.2 mg/dL (SD 94.1 mg/dL); *p* = 0.0160) in the db/db mouse model compared to the control group (748.1 mg/dL (SD 140.8 mg/dL)). Furthermore, the AP029 composition with metformin significantly reduced serum insulin levels (1.25 ng/mL (SD 1.22 ng/mL); *p* = 0.0360) in the db/db mouse model compared to the control group (2.09 ng/mL (SD 1.1 ng/mL)) (Figure 5).

### 3.6. AP029 Modulates Lipid Metabolism and Inflammation in db/db Mouse Liver

**Figure 6:**
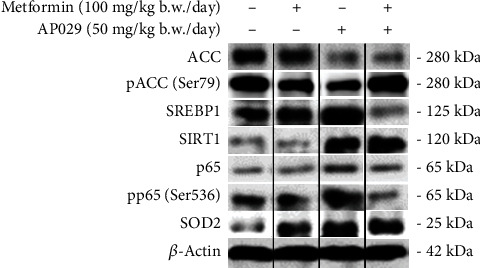
Representative western blot images showing the ACC, pACC, SREBP1, p65, pp65, SIRT1, and SOD2 protein level in the livers of db/db mouse model after treatment with metformin (100 mg/kg b.w./day), AP029 (50 mg/kg b.w./day), and AP029 (50 mg/kg b.w./day) with metformin (100 mg/kg b.w./day). Protein levels were normalized to *β*-actin.

Table 1 displays a summary of western blot densitometry data expressed as a percentage of the control and standard deviation. The liver tissue study demonstrated a notable decrease in the level of ACC after treatment with AP029 alone as well as when combined with metformin. ACC phosphorylation inhibits its activity, leading to a decrease in lipid synthesis and storage. The AP029 composition, in combination with metformin, was found to significantly increase the phosphorylation level of ACC. Additionally, the low level of ACC after AP029 with metformin treatment corresponds to the observed decrease in the level of SREBP1. SREBP1 is a transcription factor that plays a key role in regulating lipid metabolism by activating the transcription of genes responsible for lipogenesis, including fatty acid synthase and ACC. Furthermore, a significant decrease in the activity (phosphorylation) of the p65 subunit of the nuclear factor kappa B (NF-*κ*B) following treatment with AP029 in combination with metformin was observed. Analysis of liver data demonstrated a substantial elevation in the level of SIRT1 after adding AP029 with metformin (Figure 6 and Table 1).

Studies suggest that SIRT1 interacts with the p65 subunit of NF-*κ*B, inhibiting its transcriptional activity through deacetylation. The observed increase in SIRT1 level following treatment with AP029 with metformin may contribute to the observed decrease in NF-*κ*B activity and inflammation in the liver. Additionally, SIRT1 increases the expression of manganese-dependent superoxide dismutase (SOD2), one of the key regulators protecting against oxidative stress. After treatment with AP029 and metformin, the analysis revealed a significant increase in SOD2 levels (Figure 6 and Table 1).

## 4. Discussion

Black currant, chokeberry, and elderberry are abundant in polyphenols, such as anthocyanins, flavonols, and phenolic acids [31–33]. These compounds have been linked to a range of health advantages, including potential impacts on T2D [34]. Nevertheless, the existing literature on the impact of these fruits on T2D is scarce. Our study showed that AP029, the mixture of black currant, chokeberry, and black elderberry, has shown possible synergistic benefits when combined with metformin, a routinely recommended oral hypoglycemic medication for T2D. Anthocyanins, predominant in our blend, have garnered attention for their potential in mitigating T2D. The available epidemiological evidence suggests a positive correlation between the consumption of anthocyanins and a reduced risk of developing T2D, as well as improved insulin sensitivity [35]. The biochemical analysis revealed that the studied blend exhibits a high concentration of anthocyanins, with C-3-Glu, C-3-Gal, and C-3-Sam being the most prevalent. Prior research has shown that C-3-Glu and C-3-Sam have inhibitory efficacy against *α*-glucosidases and *α*-amylase enzyme activity, which are relevant to diabetes management [36]. Furthermore, research has shown that C-3-Glu has the ability to safeguard adipocytes against insulin resistance caused by H_2_O_2_ or TNF-*α* via the inhibition of c-Jun NH2-terminal kinase activation [37]. The study done by Sun et al. showed that the administration of a bayberry fruit extract high in C-3-Glu had beneficial outcomes on diabetes parameters in both in vivo and in vitro experiments [38]. The intervention effectively inhibited the apoptosis of *β*-cells, enhanced their viability, and reduced reactive oxygen species (ROS) generation inside the mitochondria [38]. The rat insulinoma INS1E cell line and mouse islets of Langerhans treated with palmitic acid further validated the observed protective impact of C-3-Glu on secretory function. This investigation demonstrated a notable drop in the expression of proteins, including BAX, which is recognized as a proapoptotic factor, as well as a decrease in apoptotic indicators such as cleaved caspase-3. Conversely, the antiapoptotic protein BCL2 was shown to be increased by C-3-Glu [39].

The mechanism by which metformin exerts its effects involves the alteration of the AMPK pathway. The activation of AMPK in hepatocytes leads to a decrease in the activity of ACC and HMGCR. This reduction in activity restricts the enzymatic processes involved in the synthesis of fatty acids and cholesterol, respectively. In addition, AMPK has inhibitory effects on the synthesis of SREBP1, which is triggered by insulin and plays a crucial role in regulating fatty acid metabolism and the process of adipogenesis. The relationship between the activation of AMPK by polyphenols and improvements in hyperlipidemia and atherosclerosis has been shown in animal models with diabetes [40]. Our findings with AP029 corroborate these effects, as evidenced by increased phosphorylation of AMPK, ACC, and HMGCR in HepG2 hepatocytes. Additionally, the downregulation of SREBP1 suggests a potential impact on lipid metabolism. The synergistic enhancement of glucose uptake observed in 3T3-L1 adipocytes further supports the activation of AMPK, aligning with studies indicating improved insulin sensitivity. This finding is consistent with the activation of AMPK, which leads to an increase in glucose uptake and a subsequent improvement in insulin sensitivity [41]. Our findings demonstrated that the coadministration of AP029 and metformin led to a significant reduction in insulin levels and fasting glucose concentrations in vivo. Further analysis of the livers of diabetic mice revealed that AP029 and metformin increased the phosphorylation of ACC, decreased the phosphorylation of p65, and decreased the total levels of SREBP1 and ACC. Additionally, the total levels of SIRT1 and SOD2, which are involved in cellular stress response and antioxidant defense, were increased. Our findings align with Anhê et al.'s data, which demonstrated that a polyphenol-rich cranberry extract reversed insulin resistance and hepatic steatosis independently of body weight loss in mice [42]. Furthermore, Li et al. found that apple polyphenol extract alleviated high-fat diet-induced hepatic steatosis in mice by targeting the LKB1/AMPK pathway [43]. Moreover, AMPK/SIRT1 signaling has been implicated in the pathogenesis of diabetes. Previous studies have found that AMPK plays a crucial role in regulating the reduction of oxidative stress mediated by SIRT1 [44]. This resonates with our findings, suggesting a shared pathway through which polyphenols may mitigate liver fat accumulation. Yang et al. investigated the hepatoprotective effects of apple polyphenols against chemical-induced liver damage, reinforcing the notion that polyphenols possess protective properties against liver injury [45]. Additionally, the study by Lee et al. on a polyphenol extract of *Hibiscus sabdariffa* L. demonstrated ameliorative effects on acetaminophen-induced hepatic steatosis, further emphasizing the potential of polyphenols in mitigating liver injury and oxidative stress [46]. Furthermore, chronic inflammation has been implicated in the pathogenesis of T2D. Inflammatory molecules and stress markers have been found in the circulation and various tissues of individuals with T2D [47]. The literature suggests that the activation of inflammatory pathways, such as the NLRP3 inflammasome, and the release of proinflammatory cytokines contribute to insulin resistance and the development of T2D [48]. Moreover, the study by Ochnik et al. evaluates the antiviral effectiveness of a blend of black chokeberry and elderberry extracts against several respiratory viruses and suggests its potential as a natural treatment option, which could be particularly beneficial for individuals with diabetes who are more vulnerable to viral infections [49]. Inhibition of inflammation and oxidative stress has been proposed as a potential therapeutic strategy for T2D. In addition to their effects on glucose metabolism, polyphenols have been shown to have anti-inflammatory properties. Chronic, low-grade inflammation is closely associated with the development of insulin resistance and T2D. Polyphenols can modulate inflammatory pathways and reduce oxidative stress, thereby improving insulin sensitivity and glycemic control [50]. Our results, coupled with these findings, underscore the diverse mechanisms through which polyphenols may exert beneficial effects on metabolic pathways.

The glucose-lowering efficacy of metformin might potentially be enhanced with the supplementation of AP029, which has been shown to enhance insulin sensitivity, reduce fasting blood glucose levels, and provide cellular protection against damage generated by oxidative stress. As a result, combining metformin with polyphenols holds the promise of achieving more robust glycemic control, a pivotal goal in diabetes management. Furthermore, the use of polyphenols alongside metformin can potentially lead to a reduction in the required metformin dosage. Lowering the dosage of metformin while maintaining therapeutic efficacy is a crucial strategy for mitigating its side effects. By doing so, individuals with diabetes may experience improved treatment tolerability and a higher likelihood of adherence, thus optimizing their overall diabetes management. Polyphenols are not only known for their role in improving glucose metabolism but also for their potent antioxidant and anti-inflammatory properties. By countering oxidative stress and inflammation, polyphenols may potentially mitigate the side effects of metformin and enhance overall treatment outcomes. However, further research is necessary in order to comprehensively comprehend the underlying processes by which these treatments operate, determine optimal dosage levels, and ascertain the potential long-term effects associated with their use. In order to validate the findings and establish the efficacy and safety of nutraceuticals in the prevention and management of T2D, it is necessary to conduct meticulously planned clinical studies and extensive research on a wider scale.

## 5. Conclusion

The study demonstrates the potential benefits of supplementing metformin with a polyphenol-rich blend, AP029, consisting of black currant, chokeberry, and elderberry extracts, in managing T2D. The synergistic interaction of these polyphenols with metformin not only aids in glucose metabolism but also provides cellular protection from oxidative stress and reduces inflammatory pathways, which are critical in the pathogenesis of T2D. These findings suggest that incorporating polyphenols could enhance the therapeutic efficacy of metformin, potentially allowing for lower dosages and improved treatment adherence by mitigating side effects. However, further extensive clinical research is needed to fully understand the mechanisms, determine the appropriate dosages, and confirm the long-term safety and efficacy of this combined therapeutic strategy.

## Figures and Tables

**Table 1:** Densitometric analyses normalized to *β*-actin ACC, pACC, SREBP1, pp65, SIRT1, and SOD2 protein levels in the livers of the db/db mouse model after treatment with metformin (100 mg/kg b.w./day), AP029 (50 mg/kg b.w./day), and AP029 (50 mg/kg b.w./day) with metformin (100 mg/kg b.w./day), presented as the percentage of control.

**Protein symbol**	**Metformin**		**AP029**		**AP029 + metformin**	
	**Mean (SD)**	***p*** ∗	**Mean (SD)**	***p*** ∗	**Mean (SD)**	***p*** ∗
ACC	105.5 (52.46)	> 0.9999	58.99 (22.89)	**0.0070**	56.14 (12.41)	**0.0008**
pACC/ACC	153.8 (65.45)	0.0978	139.4 (51.07)	0.1355	258.6 (62.98)	**0.0002**
SREBP1	110.8 (10.47)	0.1069	115.1 (33.09)	0.6991	85.87 (22.74)	0.1979
pp65/p65	111.5 (38.09)	0.5560	110.8 (51.17)	0.6547	52.91 (14.28)	**0.0031**
SIRT1	103.6 (18.00)	0.6987	117.3 (44.92)	0.7922	159.9 (40.67)	**0.0079**
SOD2	142.6 (23.32)	**0.0028**	156.0 (49.13)	**0.0260**	132.7 (25.24)	**0.0179**

∗*p* value for comparison to the control group (100%); *p* < 0.05 indicates statistically significant results.

Bold values denote statistical significance at the *p* < 0.05 level.

Data represent mean ± SD. *p* values < 0.05 were considered statistically significant.

## Data Availability

Data supporting this study are included within the article and/or supporting materials.

## Supporting Information

Supporting Information Additional supporting information can be found online in the Supporting Information section. Figure S1 In vitro adipocyte model verification. Microscopic images of 3T3-L1 preadipocytes and differentiated adipocytes at ×20 (left panel) and ×40 (right panel) magnification with scale bars indicating 100 *μ*M and 50 *μ*M, respectively. Oil Red O staining was used to confirm proper adipocyte differentiation. Figure S2 In vitro hepatocyte insulin resistance model verification. Representative western blot images demonstrating the phospho-Ser-479 Akt and phospho-Ser-9-GSK3 protein levels in HepG2 (A) and Thle-2 (B) cells cultivated in the presence of insulin (100 nM) in order to demonstrate the development of insulin resistance carried on by high glucose levels (30 mM).
