# Supplementary material for: Exploring the Potential of Ribes nigrum L., Aronia melanocarpa (Michx.) Elliott, and Sambucus nigra L. Fruit Polyphenol-Rich Composition and Metformin Synergy in Type 2 Diabetes Management
Source: J Diabetes Res. 2024 Jun 10;2024:1092462. doi: 10.1155/2024/1092462 (PMC11199064; doi:10.1155/2024/1092462)
Supplement: Supporting Information — Additional supporting information can be found online in the Supporting Information section. Figure S1 In vitro adipocyte model verification. Microscopic images of 3T3-L1 preadipocytes and differentiated adipocytes at ×20 (left panel) and ×40 (right panel) magnification with scale bars indicating 100 μM and 50 μM, respectively. Oil Red O staining was used to confirm proper adipocyte differentiation. Figure S2 In vitro hepatocyte insulin resistance model verification. Representative western blot images demonstrating the phospho-Ser-479 Akt and phospho-Ser-9-GSK3 protein levels in HepG2 (A) and Thle-2 (B) cells cultivated in the presence of insulin (100 nM) in order to demonstrate the development of insulin resistance carried on by high glucose levels (30 mM). [file 1092462.f1.docx]

**Supplementary materials:**


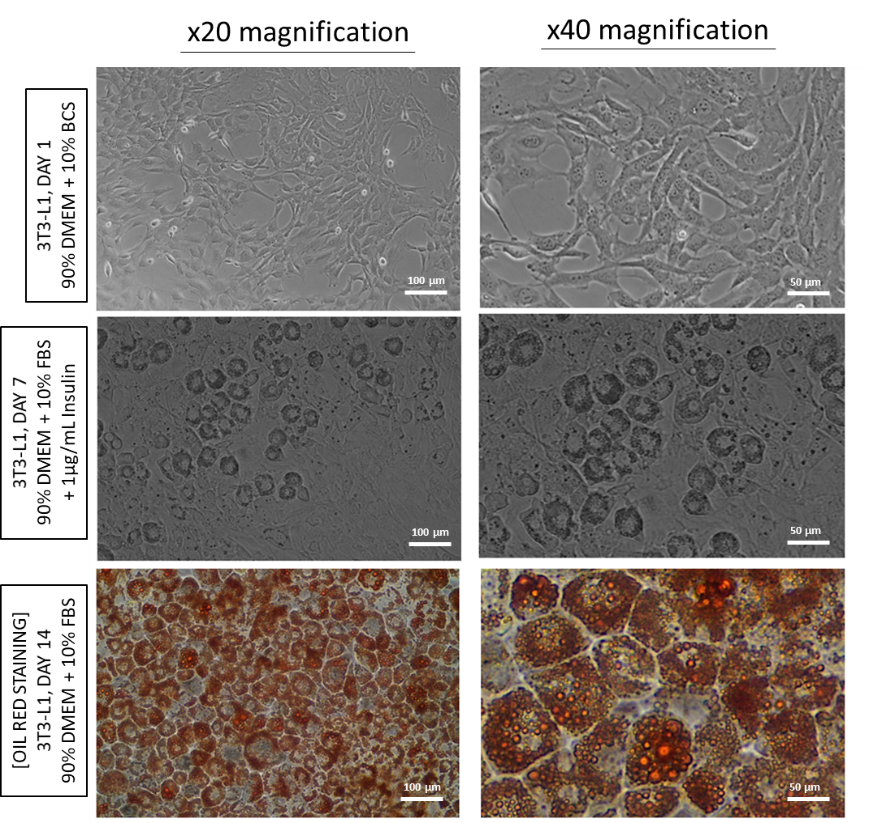


Fig. S1. *In vitro* adipocyte model verification. Microscopic images of 3T3-L1 pre-adipocytes and differentiated adipocytes at x20 (left panel) and x40 (right panel) magnification with scale bars indicating 100 μm and 50 μm, respectively. Oil-Red O staining was used to confirm proper adipocyte differentiation.


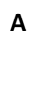

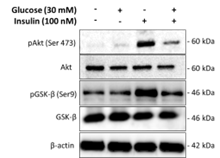

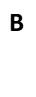

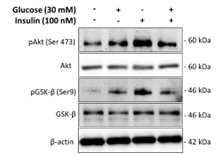


Fig. S2. *In vitro* hepatocyte insulin resistance model verification. Representative Western blot images demonstrating the phospho-Ser-479 Akt and phospho-Ser-9-GSK3 protein levels in HepG2 (A) and Thle-2 (B) cells cultivated in the presence of insulin (100 nM) in order to demonstrate the development of insulin resistance carried on by high glucose levels (30 mM).
